# Effect of Cooling/Lubrication Conditions on Machining Performance: An Experimental Investigation of 1040 Steel Under Dry, MQL, and Nano-MQL Environments

**DOI:** 10.3390/ma18174063

**Published:** 2025-08-29

**Authors:** Emin Salur, Nursena Okcu, Mehmet Erdi Korkmaz, Kübra Kaya, Rüstem Binali, Salih Bilal Çetinkal

**Affiliations:** 1 Metallurgical and Materials Engineering, Technology Faculty, Selcuk University, 42130 Konya, Türkiye; bilal.cetinkal@selcuk.edu.tr; 2 Mechanical Engineering Department, Technology Faculty, Selcuk University, 42130 Konya, Türkiye; nursenaokcuu@gmail.com (N.O.); kayakbrr@gmail.com (K.K.); rustem.binali@selcuk.edu.tr (R.B.); 3 Department of Mechanical Engineering, Karabük University, 78050 Karabük, Türkiye; merdikorkmaz@karabuk.edu.tr

**Keywords:** AISI 1040, turning, surface, cutting force, tool wear, energy consumption

## Abstract

The aim of this study is to evaluate the effect of various lubrication systems (dry cutting, MQL, and nano-MQL) on the machinability of AISI 1040 medium-carbon steel. By dispersing titanium carbide (TiC) nanoparticles into environmentally friendly sunflower oil, a new type of nano-MQL fluid was developed. Machinability parameters such as surface finish, cutting force, energy consumption, chip structure, and tool degradation were examined through scanning electron microscopy (SEM). Based on experimental observations, the use of the nano-MQL technique led to a notable enhancement in machining performance when compared to both dry and traditional MQL machining. In addition, surface roughness was substantially reduced with the nano-MQL, suggesting more effective lubrication and cooling. Reductions in cutting forces and energy consumption were also observed, indicating more efficient material removal and lower mechanical resistance. The SEM examination of the cutting tools proved the low wear rate of the nano-MQL, which exhibited less adhesion and more abrasion wear, and of dry cutting, which showed the most serious wear. Furthermore, chip morphology illustrations indicated that the chips of nano-MQL were relatively uniform and segmented, indicating superior chip breaking quality and cutting stability. The results suggest that employing TiC nanoparticles in MQL offers a clear enhancement of cutting performance in terms of process efficiency, surface quality, and tool wear. These results validate the capability of nano-MQL as an environmentally friendly and high-performance lubrication method for turning medium-carbon steels, supporting more sustainable and efficient manufacturing operations.

## 1. Introduction

Machining plays a central role in modern manufacturing, especially in producing components with high surface quality. Machining operations aim to shape a workpiece by removing material in order to achieve the desired quality, geometry, and dimensional accuracy [1,2,3]. These processes require significant amounts of energy, mainly due to the plastic deformation occurring during cutting [4]. Much of this energy is transformed into heat, resulting in elevated temperatures in the cutting area [5]. Excessive heat can negatively affect tool performance by accelerating wear, degrading tool life, and worsening the material properties of both the tool and workpiece [6]. Elevated temperatures may also increase cutting forces and trigger vibrations, which can harm surface quality and raise energy consumption as the tool loses efficiency [7]. To avoid these issues, maintaining thermal control during machining is essential [8]. Lubricating fluids are often employed to lessen friction around the cutting tool–chip interface by forming a lubricating layer, helping to lower heat and tool wear [9,10]. Nevertheless, conventional flood machining, often based on petroleum-derived fluids, raises environmental and health concerns and is generally inefficient in cooling due to poor thermal properties [11,12]. Its excessive use also leads to higher operational costs. On the other hand, dry machining eliminates the need for fluids, reducing equipment complexity and cleanup efforts [2]. Still, it comes with drawbacks, such as ineffective chip flow, accelerated tool degradation, and diminished surface integrity. To address these challenges, significant attention has been directed toward formulating energy-saving substitutes for traditional cooling and lubrication systems [13]. Minimum quantity lubrication (MQL) and its advanced variant, nano-MQL, have emerged as promising strategies in this regard [14]. These techniques aim to reduce the volume of lubricant used while improving thermal control and reducing friction during cutting operations [15].

Growing concerns about sustainability in manufacturing have led to increased focus on near-dry machining approaches, including MQL and its nanoparticle-enhanced variant. These approaches aim to lessen the negative environmental impacts associated with machining while sustaining or boosting overall process efficiency [16]. By supplying minimal quantities of lubricant as an aerosol to the machining zone, MQL methods effectively decrease fluid usage and reduce waste and management costs [17,18]. When nanoparticles are suspended in the base oil (nano-MQL), enhanced thermal conductivity and superior tribological properties are introduced, improving the performance of cooling and lubrication mechanisms at the tool–material interaction zone [1]. In contrast to conventional wet machining, environmentally friendly strategies such as MQL and nano-MQL offer multiple performance and sustainability benefits, including reductions in energy consumption, cutting forces, tool wear, and carbon emissions [19]. Furthermore, these approaches reduce operator exposure to harmful chemicals and minimize environmental contamination, contributing to cleaner and more efficient production systems [20]. By minimizing friction and heat buildup, these eco-efficient methods help cut carbon emissions, lower energy consumption, and mitigate occupational and environmental hazards tied to large-scale cutting fluid usage [21]. However, their effectiveness can vary depending on the composition of the material being processed, the nanoparticle variant, and the cutting conditions.

Despite the increasing interest in sustainable machining methods, the literature contains relatively few comprehensive studies investigating these approaches in the context of medium-carbon steels, especially AISI 1040. AISI 1040 manufacturing steel is classified as a medium-carbon steel, containing approximately 0.37–0.44% carbon [22]. This carbon content significantly influences the steel’s mechanical characteristics, enhancing its hardness and strength. In addition to carbon, the chemical composition of 1040 steel includes manganese (Mn), phosphorus (P), sulfur (S), and silicon (Si). Collectively, these elements define the mechanical performance and machinability of 1040 steel. The mechanical properties of AISI 1040 are typically expressed by its tensile strength, which ranges approximately from 580 to 700 MPa. Yield strength is another critical parameter, generally around 320 MPa for this grade [22]. These properties demonstrate that 1040 steel is well suited for applications requiring a balance of strength and durability. Its relatively high strength and good machinability allow it to be easily processed and formed into various shapes during manufacturing. Additionally, 1040 steel can undergo heat treatment to achieve different hardness levels, thereby expanding its suitability for diverse applications. Due to these characteristics, 1040 steel is widely recognized as a reliable and efficient material in the manufacturing industry for producing a variety of components. AISI 1040 steel finds widespread use across automotive, construction, and mechanical fields due to its good balance of strength and machinability [22]. It finds extensive use in mechanical manufacturing for shafts, rods, gears, bolts, and nuts. In the automotive sector, it is commonly employed in chassis parts and forged components [23]. Similarly, in agriculture, it serves as a strong and durable material for tractor parts, agricultural machinery, and other farming equipment [24].

Machinability studies on 1040 steel typically consider input factors such as feed rate, cutting rate, and depth of cutting. Output parameters often include cutting forces, surface quality, and heat generated during machining. By analyzing these parameters, optimal machining conditions can be determined to enhance process efficiency and product quality. While various studies have explored the machining performance of 1040 steel under dry and MQL conditions in milling and drilling, turning operations have remained underrepresented, especially in comparative setups involving advanced lubrication techniques. There is a substantial body of research focusing on the sustainable machining of 1040 steel, similar carbon steels, and other materials, which provides valuable insights into their behavior under various machining conditions. A graphical summary of the input and output parameters from sustainable machinability studies conducted on medium-carbon steels and various other material groups in the literature is presented in Table 1.

One drilling study [25] showed that MQL significantly suppressed torque and piercing forces in AISI 1040 compared to dry and flood conditions, offering improved dimensional accuracy and reduced tool wear. Meanwhile, turning experiments under MQL indicated reductions in cutting forces and slower flank wear progression compared to dry turning.

Tiwari et al. [26] examined the turning of AISI 1040 employing nano-Al_2_O_3_ suspended in coconut oil (nano-MQL) and reported significant improvements over dry, flood, and conventional MQL environments. Compared to dry medium, nano-MQL reduced flank wear by 53%, crater wear depth by 37%, cutting temperature by 55%, and tool vibration by 68% and improved surface finish by 54%. These results highlight the effectiveness of nanoparticle-enriched vegetable-based fluids in enhancing tool life and surface aspects.

Reports show that MQL can reduce surface irregularities, tool degradation, vibration, and heat generation while also reducing fluid handling and disposal costs. In some industrial cases, carbon footprint reductions of over 60% and substantial water savings have been reported with near-dry or MQL setups compared to traditional wet machining [27].

The work by Tuan et al. [28] focused on the hard turning process of 90CrSi material employing both MQL and MQCL techniques with Al_2_O_3_ and MoS_2_ nanofluids. Their results showed that nanofluid-assisted lubrication improved machinability significantly, increasing the highest machinable hardness by approximately 71–77%. MQCL outperformed MQL, and Al_2_O_3_ nanofluid yielded better surface roughness than MoS_2_. Among the variables studied, feed rate emerged as the key parameter influencing the quality of the surface, while fluid type and nanoparticle amount had a lesser effect.

Baldin et al. [29] evaluated the performance of two vegetable-origin and a single mineral-origin cutting fluid applied via MQL during end milling of AISI 1045 steel. They reported that while MQL notably reduced cutting temperatures and extended tool life, it had minimal impact on cutting forces, power consumption, and surface irregularities. SEM analysis revealed that abrasive wear was the predominant tool wear mechanism, accompanied by adhesive and diffusion wear. These results highlight the thermal benefits of MQL fluids without significant changes in cutting forces or energy usage.

Our previous study [30] focused on end milling AISI 1040 steel using dry and MQL techniques, demonstrating that MQL considerably decreased tool degradation, thermal load, and power consumption. Based on the ANOVA analysis, the cutting conditions accounted for 94% of the variation in power consumption and 37% in cutting temperature, while cutting speed contributed 74% to tool wear. These findings demonstrate the clear advantages of MQL over dry machining regarding sustainability and functional effectiveness.

The following points can be highlighted as a general synthesis of the aforementioned studies. Milling studies on 1040 steel have revealed that MQL can significantly reduce flank wear, cutting load, and surface quality compared to dry cutting. Drilling investigations under MQL conditions have shown reductions in torque and thermal damage, resulting in improved hole quality and tool life. Nevertheless, the majority of these works are limited in scope and do not address more advanced techniques, such as nano-MQL in turning applications. Furthermore, previous studies have predominantly used common nanoparticles, such as Al_2_O_3_ [31], MoS_2_ [32], hBN [33], or CuO [34] in their nano-MQL setups. Moreover, most nanoparticle-enhanced lubrication studies have focused on materials such as AISI 1018 [35], 1045 [36], 4140 [37], hard steels [38], and titanium-based [39] or nickel-based [40] alloys.

Considering the available literature and the authors’ present level of knowledge, the use of TiC-based nano-MQL lubrication in turning operations, especially for 1040 steel, has not been previously reported in the literature. TiC possesses high hardness, excellent thermal conductivity, and outstanding tribological characteristics, making it a promising candidate for improving the efficiency of cutting fluid applications [41]. The integration of TiC nanoparticles into an MQL technique shows promise in achieving additional reductions in tool wear, thermal load, and energy usage, while improving surface roughness and chip morphology. However, its application in the turning of AISI 1040 steel remains unexplored. Thus, this research addresses a specific and previously unexplored area by introducing a TiC-enhanced nano-MQL lubrication technique during the turning of AISI 1040 steel.

For a detailed and complete interpretation, the current research conducts an experimental comparison of dry cutting, conventional MQL, and TiC-based nano-MQL under controlled conditions. The performance indicators evaluated include the quality of the machined surface, wear on the tool flank, machining forces, power usage, and chip formation characteristics. By combining novel nanoparticle lubrication with practical turning trials on an industrially relevant material, this study contributes distinctively to the sustainable machining literature. It offers critical insights for researchers and practitioners seeking to balance environmental impact, energy efficiency, and machining performance in the high precision turning of medium-carbon steels.

## 2. Experimental Setup and Methodology

**Figure 1:**
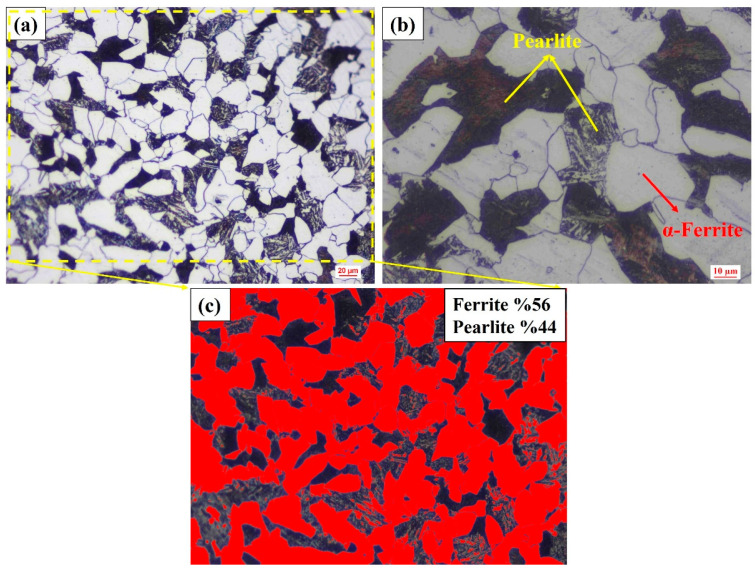
Optical micrographs of the workpiece material: (**a**) 200 X, (**b**) 500 X, and (**c**) corresponding phase areas. (Red arrow indicates ferrite, while yellow arrows indicate pearlite phase).

This section presents detailed information regarding the materials, cutting tools, machine tool, and lubricants used during machining. In addition, it provides information about the input parameters applied in machinability experiments and the measurement sensors employed for evaluating output parameters. The experimental studies were conducted on a De Lorenzo S547-8899 conventional lathe (De Lorenzo S.p.A., Milano, Italy), located within the Faculty of Technology’s Mechanical Engineering Department at Selçuk University. The test metal employed in the experiments was AISI 1040 grade steel. The elemental composition of the material is shown in Table 2, with the corresponding mechanical data summarized in Table 3. The microstructural characteristics of the AISI 1040 workpiece at different magnifications are illustrated in Figure 1, where the light-colored regions correspond to α-ferrite, and the dark regions represent pearlite. The phase analysis conducted using ImageJ software (1.53 version, Bethesda, MD, USA) is presented in Figure 1c. Based on the examination of both the microstructure and phase analysis results, the material was conclusively identified as AISI 1040 steel. In order to ensure the accurate and consistent evaluation of the output parameters, a new insert was used for each trial. The tool inserts with CCMT 09T308 geometry, manufactured by Korloy, Seoul, Republic of Korea, were used in the analysis. Additionally, the cutting tool base material is fine-grained cemented carbide containing ~8% cobalt, which provides high toughness, strength, hardness, creep resistance [42], and coating support. The cutting tool was coated with a TiAlN coating using the PVD method, which increases hardness, wear resistance, and oxidation resistance. The coating thickness was ~2–4 µm. The base hardness was ~1600 HV, and the elastic modulus was ~550 GPa, while the TiAlN coating had a hardness of ~2500 HV, an elastic modulus of ~450 GPa, and an oxidation resistance of ~800 °C. In addition, the cutting tool body was made of carbide, with a nose radius of 0.8 mm. The selected input factors for machinability assessment were cutting speed, feed, and cutting depth. The tests were conducted under three different conditions, and each input parameter was tested at two levels. These parameters were determined according to the tool maker’s recommendations and findings from a comprehensive literature review. The cutting parameters were optimized through preliminary experiments considering cutting speed, feed rate, and depth of cut. The optimal conditions were determined based on the lowest surface roughness with acceptable cutting forces, ensuring stable machining. The selected parameter levels and machining environments are summarized in Table 4.

**Figure 2:**
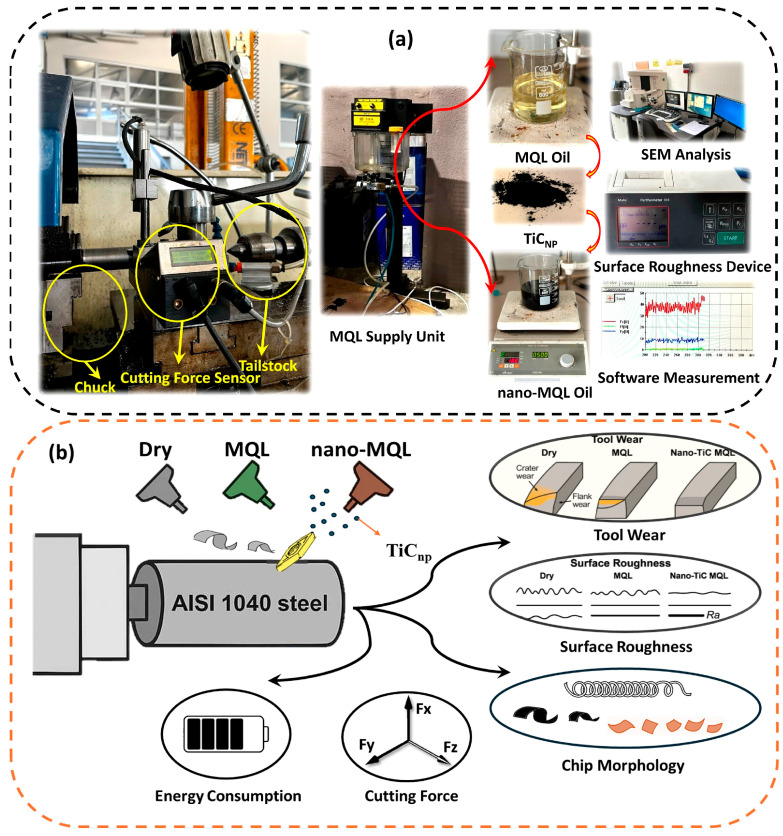
Experimental setup (**a**) and schematic illustration (**b**) of turning tests under dry, MQL, and nano-MQL conditions, showing the investigated parameters and the measurement equipment used.

Within this study, machinability performance was examined under dry, MQL, and nano-MQL machining conditions. The MQL operations were carried out using a WerteMist 15-STN device (Kar-Tes/SBH Company, İstanbul, Turkey). This MQL system was placed at a distance of 50 mm from the machining zone. The primary reason for maintaining this distance is that literature findings indicate the effectiveness of the lubrication system significantly decreases when it is positioned farther than 50 mm from the cutting zone [43]. The nozzle used in the system had a diameter of 2 mm. For lubrication, sunflower oil and TiC-enhanced sunflower oil were utilized. The transmission electron microscope (TEM) micrographs and characteristics of the TiC nanoparticles employed in this study have been reported in detail in our previous work [41]. The lubrication system delivered the cutting fluid through a 2 mm diameter nozzle at a compressor pressure of 6 bar, with a spray angle of 45 degrees. Figure 2 presents both the experimental setup (a) and its schematic representation (b), illustrating the investigated conditions of dry, MQL, and nano-MQL lubrication, together with the analyzed machinability aspects and the equipment employed in this study.

In this study, tool wear, surface quality, cutting force, and chip characteristics were systematically evaluated. Flank wear observations were obtained using an Zeiss Evo LS10 SEM (Carl Zeiss Microscopy, Cambridge, UK). Additionally, EDX was employed to provide regional and mapping analyses of the worn surfaces. Chip formation during cutting was also examined using the same SEM device. The surface roughness of the workpiece was measured after every trial using a perthometer (Mahr Co., Ltd., Göttingen, Germany), featuring a detection limit of 150 μm. In each test, surface measurements were performed five times on the machined area. To determine the representative surface roughness (Ra) values, a cut-off length of 0.8 mm and a tracing length of 5.6 mm were selected, and the Ra values were measured according to the ISO 4278 standard. The final roughness value was obtained by eliminating the highest and lowest values and averaging the remaining three measurements. During machining, the cutting force for each experiment was recorded using a cutting force measurement sensor (model: Telc, Frankfurt, Germany).

Data collected from the force sensor were recorded using XKM2000 software (Germany). Energy consumption refers to the total amount of energy consumed during a specific machining operation. It can be expressed in various units, such as kilowatt-hours (kWh), joules (J), or calories (Cal). Among the various fields in which energy consumption is monitored, it is most prominently observed in the manufacturing industry, particularly in machining operations. The main reason for this is that machining processes are not only responsible for finishing but also for transforming raw materials into final products. High cutting speeds result in increased frictional forces at the cutting zone, which consequently lead to the generation of heat-dissipated as energy. In summary, cutting speed and energy consumption are directly proportional, making the measurement of energy consumption critical in machinability studies. This paper investigated the amount of energy consumed during turning under multiple cutting conditions and lubrication environments. This objective was accomplished by employing a KAEL Multiser 02 PC TFT Network Analyzer (Kael Elektronik Mühendislik Ltd. Şti., İstanbul, Turkey). To enable accurate measurement in the high-capacity three-phase lathe system (rated above 30 A), three 60/5 A capacity current transformers were connected. These transformers were used to reduce the current to measurable levels while preserving accuracy. The detailed procedure used for measuring energy usage is available in our earlier research [44].

## 3. Results and Discussion

### 3.1. Surface Roughness

**Figure 3:**
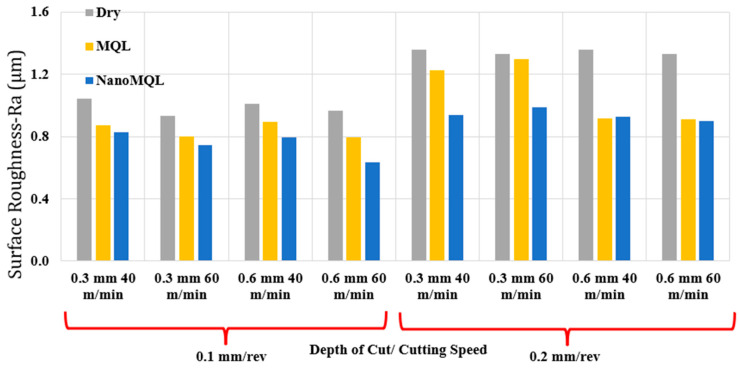
Correlation between surface quality and cutting parameters and cutting environments.

Among the various parameters, surface roughness is the primary output parameter in machinability evaluations, serving as a key indicator of surface integrity [45,46,47]. Optimizing surface roughness requires the appropriate selection of machining parameters, as it is affected by different factors, including the tool, the mechanical properties of the workpiece material, and the vibrations generated during the cutting operations [48,49,50,51]. Figure 3 demonstrates the graphical representation of roughness the measurements during the experimental trials conducted in this study. The optimum surface roughness was achieved under the conditions of a feed rate of 0.1 mm/rev, a cutting speed of 60 m/min, a depth of cut of 0.6 mm, and under a nano-MQL environment. Conversely, the poorest surface quality was recorded at a feed rate of 0.2 mm/rev, a cutting speed of 40 m/min, a depth of cut of 0.3 mm, and under dry cutting conditions. When comparing different lubrication environments, dry cutting consistently resulted in the highest surface roughness across all parameter combinations, while nano-MQL yielded the lowest values. These findings indicate the necessity of using a cutting fluid to achieve optimal surface finishes. The superior performance of nano-MQL in reducing surface roughness is attributed to its enhanced lubrication, effective heat dissipation, and the cushioning effect introduced by nanoparticles, which collectively reduce friction and fluctuations in cutting forces [38]. Similar findings are reported in [44,52]. When the graph is further analyzed, an increase in feed values is found to cause a corresponding increase in surface roughness values. This observation is consistent with findings in the literature [53,54,55]. Higher feed rates result in greater chip removal rates, which adversely impact the surface quality and increase roughness levels [56]. A noticeable decrease in surface roughness was observed with higher cutting speeds. The observed trend results from the thermal conditions at increased speeds, which facilitate the plastic deformation process in the workpiece [57]. However, under the specific conditions of a 0.2 mm/rev feed rate, a 0.3 mm depth of cut, and both MQL and nano-MQL environments, a rougher surface was obtained at higher cutting speeds. This observation is consistent with [58,59] and can be explained by micro-fractures at the tool tip and accelerated tool wear at higher speeds [60,61]. Varying cutting depths did not result in significant irregularities in surface roughness, suggesting that this parameter has a relatively minor influence compared to the feed rate and cutting speed [62]. Finally, the relative increase in surface roughness under dry cutting conditions was calculated as 45.60%, compared to 62.62% under MQL and 55.93% under nano-MQL.

### 3.2. Energy Consumption

**Figure 4:**
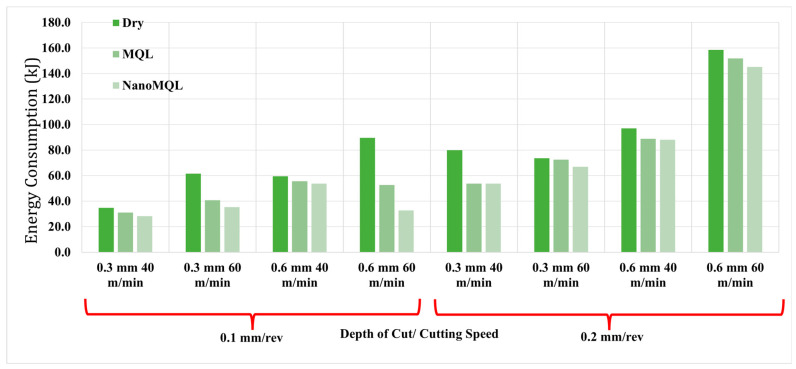
Variation of energy consumption with respect to machining parameters and cutting environments.

Minimizing energy consumption in machining processes offers significant economic benefits and has become a key consideration in sustainable and green manufacturing practices [63,64]. Accordingly, controlling energy use and maintaining it at the lowest possible levels during production has emerged as a prominent research focus. Figure 4 displays the graph of energy consumption recorded during the experimental trials conducted in this study. The lowest energy consumption was observed under the machining conditions of a 0.1 mm/rev feed rate, 40 m/min cutting speed, 0.3 mm depth of cut, and nano-MQL as the lubrication method. In contrast, the highest energy consumption was recorded at a feed rate of 0.2 mm/rev, a cutting speed of 60 m/min, a depth of cut of 0.6 mm, and under dry cutting conditions. The higher energy demand observed in dry machining can be attributed to the chip formation mechanism. In the absence of lubrication, the chip pressure in the cutting zone increases, resulting in longer and more resistant chip formation. The application of cutting fluids, particularly under nano-MQL conditions, reduces this pressure and promotes shorter chip segmentation, thereby decreasing energy consumption [65]. An increasing trend in energy consumption was recorded with rising feed rates. This can be explained by the greater volume of material removal and the larger contact area between the tool and workpiece, which requires higher power for chip evacuation as the feed increases [66]. This pattern is also consistent with findings reported in previous studies [67,68]. Energy consumption demonstrated varying behaviors, with changes in cutting speed. A decrease in energy consumption with increasing cutting speed, as observed in certain trials, is also supported by [69]. This may be due to reduced machining time and more efficient chip evacuation at higher speeds, which together contribute to energy savings. However, in other experimental sets, increased cutting speed led to higher energy consumption. This may be attributed to elevated cutting temperatures that accelerate tool wear, thereby reducing tool efficiency and increasing cutting resistance, factors which collectively raise energy demand [70]. Similar to cutting speed, energy consumption exhibited both decreasing and increasing trends with variations in depth of cut. The decline in energy consumption with increased depth of cut, as noted in [69], is attributed to enhanced material removal rates, which improve energy efficiency under constant feed and speed conditions. Conversely, in some conditions, increased depth of cut resulted in higher energy consumption due to the enlarged cross-sectional area of the cutting zone, which elevated the cutting force and mechanical load on the tool, thereby increasing the overall energy requirement [71].

### 3.3. Cutting Force

**Figure 5:**
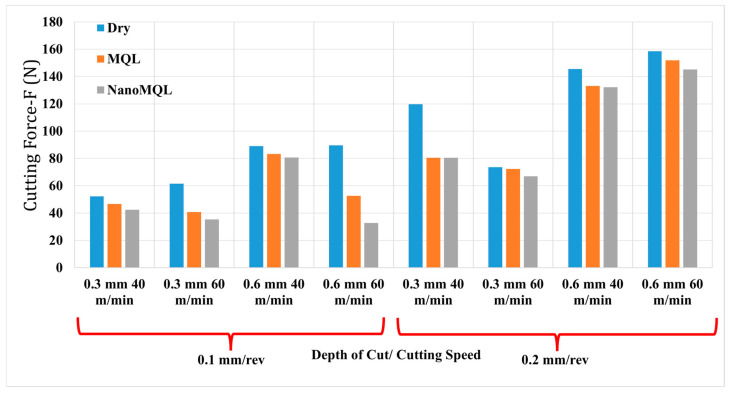
Variation of cutting force with respect to machining parameters and cutting environments.

Cutting force is a fundamental machinability output parameter that arises from the mechanical motion of the cutting tool and its interaction with the workpiece. In machining operations, low cutting forces are desirable, as they indicate that the material can be machined with less resistance, an essential criterion for improved process efficiency. Within the scope of this study, the evaluated cutting force corresponds to the main cutting force, F_c_. Figure 5 graphically presents the cutting forces measured in the experiments. As illustrated, dry cutting conditions produced the highest cutting forces across all parameter combinations, while the lowest forces were consistently measured in the nano-MQL environment. These findings align with those reported in previous studies [72,73]. The effectiveness of the nano-MQL environment in minimizing cutting force is attributed to the presence of nano-enhanced lubricants, which form a thermally stable, thin oil film in the cutting zone. This film significantly reduces friction and adhesion between the tool and the workpiece, facilitating smoother cutting and thereby lowering the required cutting force. Among the machining parameters, feed rate and depth of cut are the most influential factors that affect cutting force [74]. An increase in feed rate elevates the power demand for chip deformation, thereby raising the cutting forces [75,76]. This trend is widely acknowledged in the literature [77]. The relationship between cutting speed and cutting force exhibits a more complex pattern. In some instances, higher cutting speeds lead to increased cutting forces, potentially due to chip accumulation in the cutting zone [78]. Conversely, a reduction in cutting forces at elevated cutting speeds may result from decreased adhesion of the work material to the tool, diminished tool wear, and more stable cutting conditions [79]. Similar dual trends are observed with respect to the depth of cut. In certain cases, an increase in cutting depth reduces cutting force. This can be explained by the enlargement of the chip cross-sectional area, the formation of micro-cracks along the cutting path, and improved chip evacuation, all of which contribute to easier material removal and reduced cutting load [80]. However, in other scenarios, a greater depth of cut leads to increased cutting forces due to the larger contact area and higher mechanical stress exerted on the tool. This outcome is documented in the literature [81].

### 3.4. Tool Wear

**Figure 6:**
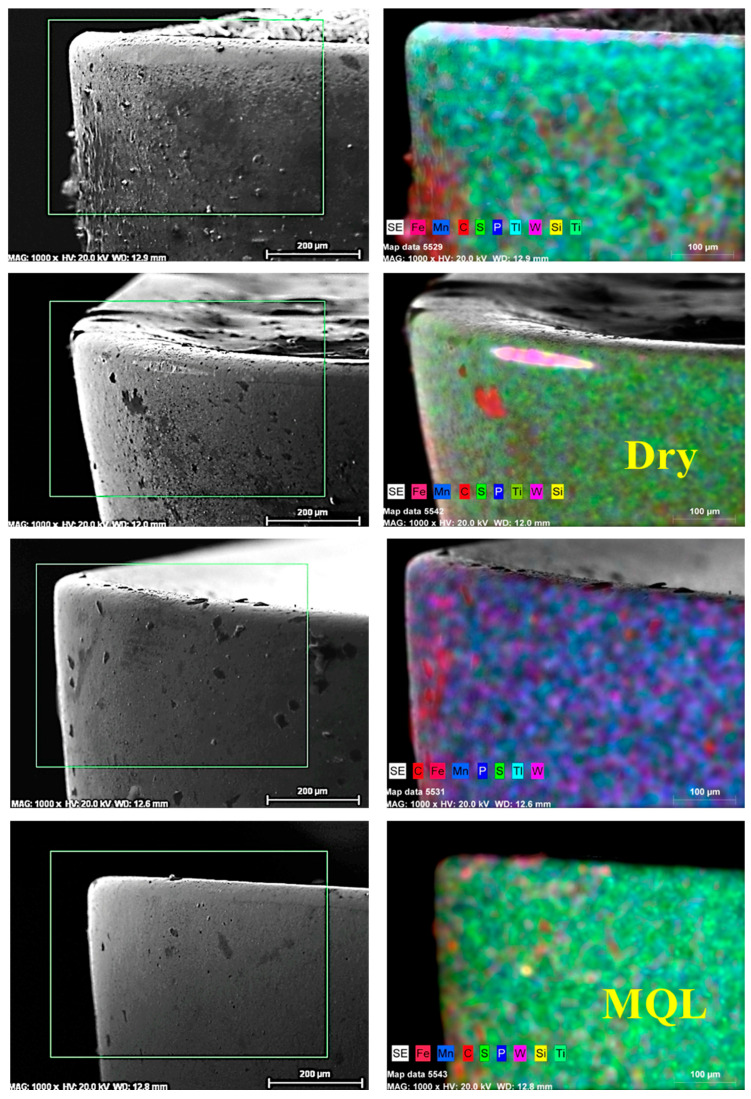

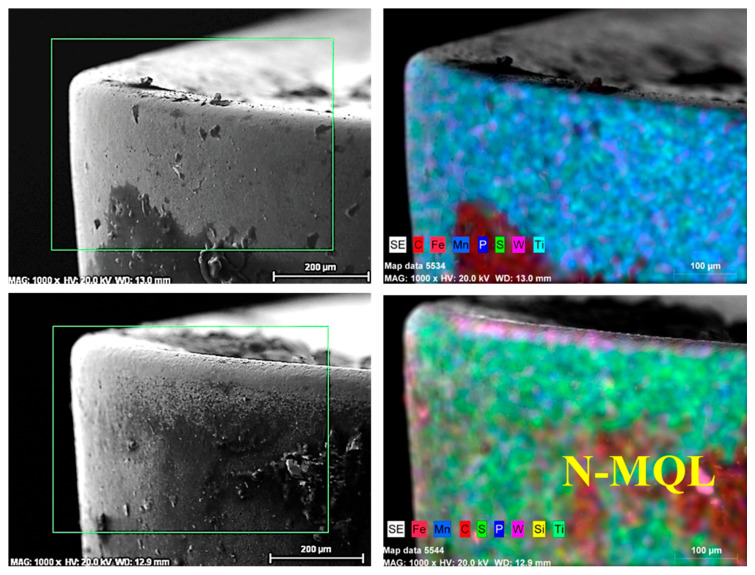
SEM images of cutting inserts with respect to cutting environments.

Tool wear is a significant factor that influences machining performance, including surface roughness, form accuracy, and production costs [82]. In this work, the direct measurement of tool life or quantitative wear parameters (flank wear width or crater depth) was not carried out; however, the wear mechanisms were qualitatively assessed by means of SEM [45]. Scanning electron microscope images of the cutting inserts employed under dry, MQL, and nano-MQL conditions are presented in Figure 6.

The SEM observations indicated distinct differences in the wear modes depending on the lubricated conditions. In dry machining, the worn features of the cutting inserts included abrasive marks, chipping, and adhesive material on the rake face. These defects indicated that insufficient lubrication resulted in higher friction and thermal loads, which facilitated both abrasive and adhesive wear [83]. Tool edges also exhibited microfractures and BUE due to high stress and high temperature conditions of hard alloy under dry cutting. The use and composition of the cutting fluid were found to influence BUE formation. In comparison, the inserts tested under MQL conditions showed relatively smoother wear areas. Although some abrasive wear marks and material adhesion on the edge were still observed, the degree of wear was visually lower than in dry cutting. The lubricating MQL system may have formed a friction-reducing film between the tool and the chips; consequently, thermal and mechanical stresses could have been relieved, due to the properties of the thin oil film. The rake and flank faces exhibited reduced wear, and the tribological properties were improved.

The best surface quality of the tool inserts was obtained with nano-MQL lubrication. The SEM observations revealed less wear than under any other cutting conditions, with a cleaner rake face and sharper cutting edges. Only slight wear, caused by adhesive and abrasive mechanisms, was observed. The addition of TiC nanoparticles to the cutting fluid may have resulted in better lubrication and more efficient heat dissipation during machining. These nanoparticles act as solid lubricants or rollers, creating a protective tribo-layer that decreases tool–chip interaction and prevents severe wear. Despite the lack of quantitative tool wear analysis, the SEM examinations clearly indicate that cutting tools have better protection against wear in the nano-MQL approach compared to MQL and dry cutting. These findings are indicative of the ability of lubrication systems to prolong tool life and maintain stable machining performance.

### 3.5. Chip Morphologies

**Figure 7:**
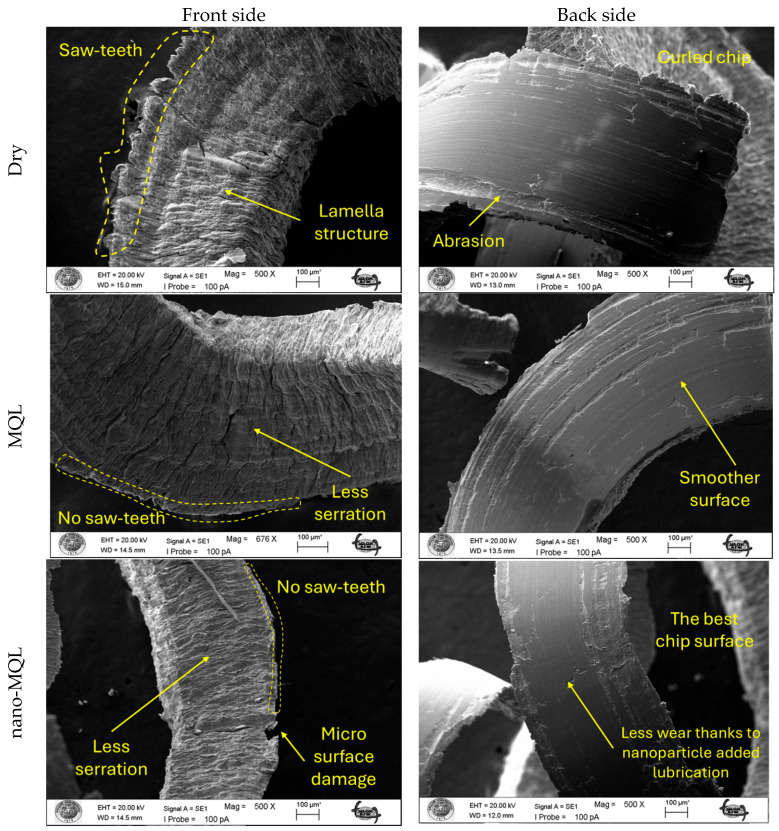
SEM images of chips from the front and back sides under different cutting environments.

The chip morphology can reveal the material removal mechanism, the thermal and mechanical conditions in the cutting zone, and the performance of the lubrication method [8,84]. The chips produced during the turning of AISI 1040 steel were collected under dry, MQL, and nano-MQL conditions and analyzed using SEM. Representative SEM images of the chip forms produced under the lubrication media are depicted in Figure 7.

Dry cutting conditions exhibited poor chip breakability, resulting in mostly long, uninterrupted, curled chips. These chips had saw-like edges and nonuniform surfaces, which indicated an unsteady cut and high shear zone temperature. The absence of lubrication may lead to excessive heat generation and high friction at the tool–chip interface, which in turn results in more severe plastic deformation but less effective chip control. Chip adhesion and tear were also observed, which could influence built-up edge formation and tool wear. In MQL, the chips were shorter and more segmented than those produced under dry cutting conditions. The chip surfaces were smoother, with fewer serrations and deformation marks. This enhancement in chip morphology is attributable to the lubricating film supplied by the MQL system, which led to a noticeable reduction in frictional forces and cutting temperatures [31,85]. Therefore, the chips were easier to rupture and detach, which facilitated more stable machining and reduced mechanical load on the cutting edge. Under nano-MQL, the chips exhibited the best morphology. They were well proportioned, short, and composed of relatively few segments, but there was a little surface damage. It was observed that the cutting edges of the chip were well-designed, and only fine chips were generated, which indicates that the level of plastic deformation was controlled, and the heat dissipation was efficient during cutting. The suspended TiC nanoparticles in the base oil probably contributed positively to cooling and lubrication behavior, thus leading to a better chip breakability and lower thermal stress inside the cutting region. The improved chip removal also kept the insert face dry and clean, thus lowering the percent entanglement of the chips to reduce the wear and untimely maintenance of the CNC machine. In conclusion, the observation of chip shapes clearly underlines the importance of lubrication in chip formation. Long, irregular chips in the cutting zone during dry cutting can result in poor tool life and/or surface finish. MQL yielded better chip control, whereas nano-MQL produced the most favorable chip forms, indicating its efficiency and sustainability in machining.

### 3.6. Machining Efficiency

In this study, machining efficiency was calculated based on cutting speed, feed rate, depth of cut, surface roughness, and cutting force parameters using the formula presented in Equation (1). In the formula, $V_{c}$ represents the cutting speed, f the feed rate, $a_{p}$ the depth of cut, $R_{a}$ the surface roughness, and $F_{c}$ the cutting force.

(1) $$MEI=\frac{V_{c}\times f\times a_{p}}{R_{a}\times F_{c}}$$

The aim was to investigate the influence of different cutting environments on machining efficiency. By applying the formula in Equation (1) to the experimental data, the MEI values presented in Table 5 were obtained. The experiments conducted under different lubrication conditions revealed that machining efficiency (MEI) values varied significantly. Under dry conditions, the average MEI was 2.8922%, whereas it increased to 4.4622% with MQL and reached 6.1605% with nano-MQL. These results demonstrate that the type of lubrication plays a crucial role in enhancing machining efficiency.

In particular, the nano-MQL application achieved the highest MEI value, offering more effective and faster production during machining. The examination of the standard deviation values indicated that the dry condition was more consistent but less efficient, while the MQL and nano-MQL conditions provided higher efficiency, with increased variability between measurements. This finding highlights that nanofluid-assisted MQL applications, although capable of delivering high efficiency, require careful process control.

## 4. Conclusions

The present study experimentally investigated the turning of AISI 1040 steel under three lubrication conditions, including dry cutting, traditional MQL, and the nano-TiC/MQL technique. The findings are helpful in comparing advanced lubrication strategies for enhanced machining performance and eco-friendly manufacturing.

Surface quality observations showed that the best surface finish was achieved with the nano-MQL. This enhancement is ascribed to the improved lubrication and cooling influence provided by the TiC nanoparticles, which can alleviate tool–workpiece friction and thermally destabilize. Dry cutting always yielded the worst surface roughness, which was caused by both the absence of lubrication and the surface damage and rough profile produced by excessive heat generation.

With regard to energy consumption, nano-MQL performed better than the two other methods. This nanoparticle-enhanced lubrication improved chip segmentation and heat conduction, which in turn reduced cutting resistance and the power required for machining. Dry cutting consumed the greatest amount of energy overall due to higher cutting resistance and ineffective chip evacuation.

The cutting force test also proved the efficacy of nano-MQL. The presence of TiC nanoparticles facilitated the transformation of a thin protective lubricating layer formed between the tool and chip surfaces, which effectively decreased adhesion and friction. Consequently, the cutting force was lower than that observed in MQL and dry cutting. These lower forces lead not only to better energy efficiency but also to reduced mechanical stress on the cutting tool.

SEM observation served as an important verification of tool wear characteristics. The inserts under dry conditions revealed different wear behaviors, such as abrasion, adhesion, and edge chipping. MQL provided moderate protection, whereas nano-MQL resulted in the lowest wear, characterized by smoother surface and minimal material damage, thereby offering the best wear resistance.

The beneficial effects of nano-MQL were also revealed through chip morphology analysis. The chips formed under nano-MQL were short, segmented, and uniform in shape, indicating stable cutting and more effective thermal control. On the other hand, the long and irregular chips generated under dry cutting indicated unstable cutting conditions and poor chip-breaking capability.

The assessment of machine efficiency across different cutting environments revealed that the highest efficiency was achieved under nano-MQL conditions, followed by MQL and dry cutting conditions, which exhibited progressively lower performance.

The MEI calculations clearly demonstrated the significant impact of lubrication conditions on overall machining performance. Among the tested environments, nano-MQL provided the highest efficiency, with an MEI of 6.1605%, followed by MQL at 4.4622% and dry cutting at 2.8922%. The improvements observed under nano-MQL conditions can be attributed to reductions in cutting force and surface roughness, leading to more efficient material removal. These findings highlight the importance of advanced lubrication techniques in enhancing machining efficiency, particularly in sustainable and high-performance manufacturing processes.

The overall results suggest that TiC-based nano-MQL has great potential for enhancing the machining characteristics of AISI 1040 steel compared to conventional methods. This lubrication method improves surface finish, reduces energy consumption and cutting forces, and minimizes tool wear. Such results are not only helpful in achieving good machining efficiency but also contribute to green and sustainable manufacturing. These findings provide a basis for future studies on nanoparticle-assisted lubrication systems in turning and other machining operations.

## Figures and Tables

**Table 1:** The input and output parameters and corresponding result evaluations in the machining of workpiece materials.

Input Parameters	Output Parameters	Result Evaluations
Cutting Depth	Surface Roughness	Graphical
Cooling Medium	Cutting Force	Machine Learning
Cutting Speed	Tool Wear	SEM
Feed Rate	Energy Consumption	Modeling

**Table 2:** The elemental composition of AISI 1040.

C %wt.	Mn %wt.	P %wt.	S %wt.	Si %wt.
0.37–0.44	0.60–0.90	~0.04	~0.05	0.15–0.30

**Table 3:** The mechanical properties of AISI 1040 steel.

Properties	Value
Tensile Strength	580–700 MPa
Yield Strength	320–530 MPa
Elongation at Break	13–20%
Brinell Hardness	160–180 HB
Shear Strength	350–390 MPa
Elastic Modulus	190–210 GPa
Poisson Ratio	0.27–0.30%
Thermal Expansion Coefficient	12 µm/m·°C
Thermal Conductivity	51 W/m·K
Density	7.8 g/cm^3^

**Table 4:** Cutting parameters and environments.

Description	Value
Cutting speed (m/min)	40, 60
Feed rate (mm/rev)	0.1, 0.2
Cutting depth (mm)	0.3, 0.6
Cutting environment	Dry, MQL, and nano-TiC/mQL

**Table 5:** MEI results under different cutting environments.

Cutting Environments	MEI Values
Dry	2.8922%
MQL	4.4622%
Nano-MQL	6.1605%

## Data Availability

The raw data supporting the conclusions of this article will be made available by the first author on request.
